# More Experience, Less Loneliness? Exploring the Effect of Experiential Purchases on the Alleviation of Loneliness

**DOI:** 10.3389/fpsyg.2021.581183

**Published:** 2021-08-09

**Authors:** Bingcheng Yang, Hongyan Yu, Yu Yu

**Affiliations:** Business School, Sun Yat-sen University, Guangzhou, China

**Keywords:** loneliness, experiential purchases, material purchases, relational enhancement, social nature

## Abstract

Over the past few decades, researchers have explored the effects of experiential purchases and material purchases on happiness and provided a range of evidence that consumers yield greater happiness from experiential purchases compared with material purchases. However, limited research is known about the relationship between these two types of purchases within the broader context of negative emotion. Specifically, the current research focuses on the effect of experiential purchases on loneliness alleviation to replenish this research stream. Three experiments were conducted to explore the effect of experiential purchases (vs. material purchases) on alleviating loneliness. The results showed that experiential purchases have a stronger effect on loneliness alleviation than material purchases, which is mediated by relationship enhancement. In addition, purchases of social nature moderate the effect of experiential purchases on loneliness. Social experiential purchases lead to a higher degree of relief of loneliness. On the contrary, for the solitary experiential purchases, the effect of experiential purchases on loneliness is less tight. The current research supplements the research on negative emotions of experiential purchases and expands the research area of experiential purchases, which also provides new insights into coping strategies of loneliness.

## Introduction

With the development of information technology and social networks, people are increasingly connected digitally, but the prevalence of loneliness seems to be rising (Habibi et al., 2018). According to a European Commission (2018) report, more than 75 million European adults meet with family or friends only once a month, and around 30 million European adults frequently feel lonely. As a negative effect in response to a lack of social connections, 72% of Americans also reported experiencing loneliness (Muyan et al., 2016). Another report also reveals that young people are more likely to feel lonely than older people, according to a survey of 55,000 people worldwide (Manchester Institute of Education, 2018). Due to more self-isolation and lack of social interaction during the COVID-19 pandemic, people are threatened with a higher sense of loneliness than normal (Killgore et al., 2020).

Loneliness refers to a subjective perception of social isolation under the discrepancy between the expected social connection and the actual social connection (Cacioppo et al., 2009). Loneliness has a severe negative impact on psychological well-being, such as triggering anxiety over concern, frustration reactivity, the occurrence of depressive symptoms, and physical health hazards, such as cardiovascular diseases and reduced immunity (Deckx et al., 2018). Due to the prevalence and negative impact of loneliness, it is important to develop strategies that may help decrease its adverse impact. Given that loneliness is a negative state of perceived social relationships (attachments) that do not attain desired levels, specific consumption can be one potential strategy for relieving loneliness, as consumption can fulfill the goals of social acceptance and a sense of belonging (Mead et al., 2010). Different types of consumption also have an important impact on the emotions and cognition of consumers due to the differences in intrinsic nature. However, research on the connection between loneliness and consumer behavior has not been addressed until recently as a positive intervention for loneliness (Pieters, 2013; Wang et al., 2021). In the current research, we examined the effects of the two most widespread purchase-experiential purchases and material purchases on loneliness.

Experiential purchases, such as watching a movie or a football game, refer to purchases for the purpose of experience, while material purchases refer to the purchase of physical objects for preservation or possession, such as jewelry or furniture (Van Boven and Gilovich, 2003). Existing serious of literature have helped clarify why people derive more happiness from experiential purchases than material purchases. For instance, relative to their material purchases, individuals interpreted their experiential purchases as being more closely connected to their sense of self (Carter and Gilovich, 2012), invoking less rumination (Carter and Gilovich, 2010), and more willing to share and communicate (Howell and Hill, 2009; Kumar and Gilovich, 2015). Experiential purchases also have a higher conversational value, which helps afford greater happiness of consumers than do objects (Bastos and Brucks, 2017). Recent works have also provided substantial evidence that experiential purchases can satisfy individual relationship needs and reduce the adverse effects caused by individual social comparison (Van Boven and Gilovich, 2003; Gilovich et al., 2015; Kumar and Gilovich, 2016), which may have an important implication for alleviating loneliness. However, the significant difference between these two types of purchases seldom sheds light on the relief of negative emotions (such as loneliness). Seeking to extend previous work, the present study examines the effects of experiential purchases on an important negative emotion—loneliness.

To address this question, the current research investigates how and why experiential purchases (vs. material purchases) can more effectively reduce loneliness and explore the mechanism of experiential purchases on the relief of loneliness through three experiments. As experiential purchases are more related to others, which helps to compensate social connection than material purchases, and provides the possibility for the alleviation on loneliness. The current research has an important enlightening contribution to existing literature and practice. On the one hand, this study complements the research on experiential purchases in negative emotions and expands the scope of research on loneliness. On the other hand, the results also provide new insights into the coping strategies of loneliness and that the essential nature of purchases is an enlightening interpretation for relieving loneliness and improving welfare. Next, we review the theoretical background, develop the hypotheses, and report three experiments that examine our propositions.

## Theoretical Background and Hypothesis

### Experiential Purchases and Material Purchases

The distinction between material and experiential purchases was first introduced by Van Boven and Gilovich (2003). Material purchases are defined as the consumption for the purpose of purchasing material property (such as buying clothes, jewelry, or electronic products, etc.), which emphasizes the possession and preservation of the product. Distinctively, experiential purchases are defined as the consumption for the purpose of obtaining a life experience (such as travel, dining, and concerts, etc.) (Van Boven and Gilovich, 2003), which emphasizes the intangible nature of experience. It is worth noting that the boundary between experiential purchases and material purchases is not always so strict; they are more considered as two ends of the continuum (Van Boven and Gilovich, 2003; Carter and Gilovich, 2012). Some purchases, such as a video game console or a bicycle, may both be material and experiential. Although they are tangible products, people purchase them mainly for their experiential attributes. For those that cannot be clearly distinguished, the original purpose of the purchase may serve as the division of indicators (Van Boven and Gilovich, 2003). The research on experiential purchases and material purchases originated from psychology, where people are questioning about the source of happiness. A series of studies have documented that, compared with material purchases, experiential purchases give rise to more enhancement of subjective well-being (Carter and Gilovich, 2010, 2012; Caprariello and Reis, 2013; Lee et al., 2018) and more resistance to hedonic adaptation (Pchelin and Howell, 2014), which are also called “experiential advantage.” Regarding the reasons for the positive effect of experiential purchase on happiness, previous research has also conducted in-depth exploration. Dunn et al. (2011) claimed that out of the self-protection of memory, consumers have a more active memory construction for experiential purchases, which will cause the intent of consumers to interpret and evaluate past experiences more positively, thereby increasing their sense of happiness. Bastos and Brucks (2017) documented that compared with material purchases, experiential purchases contribute to more happiness due to the higher conversational value, recognition of self-identity, and close social relationships. In addition, compared with material purchases, experiential purchases are less likely to cause social comparisons, which can reduce the negative impact of social comparisons and lead to higher levels of consumer happiness (Carter and Gilovich, 2014).

Experiential purchases are more conducive to interpersonal conversations and also, inherently, more social than material possessions, which ought to be more beneficial for the satisfaction of relatedness need (Nicolao et al., 2009; Kumar and Gilovich, 2016). As mentioned above, previous research has paid more attention to the significance of experiential purchases on positive emotions, such as happiness, etc.; the effect of experiential purchases on negative emotions is limited. However, experiential purchases may also serve an important role for negative emotions. Lin (2018) found that experiential purchases and phrasing were less likely to be perceived as showing off on social media and thus may triggered more benign envy (motivates individuals to improve themselves and achieve the same success as others) than malicious envy (harm and belittling others and leads to negative social comparisons). Rosenzweig and Gilovich (2012) claimed that experiential purchase decisions are more likely to lead to regrets of inaction (missed opportunities that could have been done but was not), while material purchase decisions are more likely to generate regrets of action (remorse of a buyer that has not been done, but has done). Addressing to this stream, the current research seeks to explore the relationship between experiential purchases and loneliness, and broaden the research of the role of experiential purchases in the alleviation process of negative emotions.

### Experiential Purchases and Loneliness

In recent years, the research on loneliness has become more and more popular and crucial. As a common kind of distressed feeling experienced when social needs are not satisfied (Al-Yagon, 2008), loneliness is increasingly recognized as a non-negligible public concern (Muyan et al., 2016). Humans are essentially social animals. Such kind of biological strategy requires individuals to maintain a deep connection with other individuals as the fundamental factor of nearly all human striving. Loneliness occurs when actual experiences of interactions and emotional connections of individuals are lower than their expectations for interpersonal relationships (Russell et al., 1980; Luo et al., 2012). In general, loneliness is described as an intensive subjective feeling rather than an objective state. As a consequence of social isolation, loneliness is manifested by cutting ties with social groups that potentially raise the threat of losing connections with social interaction and existential value (Cacioppo et al., 2009). Loneliness is often accompanied by unhealthy emotional reactions. Particularly, it drives a series of physical and mental health problems, such as depression (Peplau and Perlman, 1982), health disorders (Cacioppo et al., 2002), mental loss (Masi et al., 2011), alienation, and even adjusted all-cause mortality (Creemers et al., 2012; Luo et al., 2012). Seeking to decrease individual loneliness, perception has become a non-negligible issue for the academia and society.

A considerable research has focused on how external interventions can mitigate loneliness (Cacioppo et al., 2015; Muyan et al., 2016; Deckx et al., 2018), but limited studies have shed light on the coping strategies of purchase types in the face of loneliness. Previous studies have explored a range of associations between loneliness and consumption behavior (Lastovicka and Anderson, 2014). For example, Pieters (2013) claimed that lonely individuals prefer materialistic products; thus, materialism may, in turn, reinforce individual loneliness and create a negative cycle. Jing et al. (2012) found that lonely consumers prefer minority-endorsed products, while non-lonely consumers prefer majority-endorsed products. To regain social connections, lonely people may prefer interaction with high anthropomorphic hedonism (Feng, 2016). A recent study also revealed that lonely individuals may prefer more conspicuous consumption, which is driven by mating motivation (Liu et al., 2020). Noticeably, previous studies mainly focused on the compensation of specific consumption types of loneliness, while ignoring the essence inherent in specific purchase types. As the most widespread type of consumer behavior, the essential nature between experiential and material purchases may also have a discriminative effect on loneliness. As Lastovicka and Sirianni (2011) claimed that material possession is closely related to a lack of social affiliation and loneliness, while experiential purchases may foster social affiliation and reduce the loneliness that experiential purchases involve social interactions, e.g., sharing experiences, and conversations, etc. (Chan and Mogilner, 2016; Yamaguchi et al., 2016; Bastos, 2020).

In the current study, we propose that experiential purchases may serve as a potential coping strategy for loneliness. We consider that experiential purchases may alleviate loneliness for the two following reasons. On the one hand, the embedded social nature of experiential purchases may promote stronger social connections than material purchases. Individuals with the same experiences (e.g., being a member of a club) are more socially relevant and connected than those with the same product (e.g., owning the same brand of car) (Kumar and Gilovich, 2016). Compared with material purchases, experiential purchases tend to present a higher frequency of interaction with others in the process of consumption (Gilovich et al., 2015), which are more related to alleviate loneliness. Travel, adventures, watching movies, and other experiential purchases exhibit inherent features of social connections, which can be considered as a process of consumption that is more conducive to promoting human interactions and social relationships (Van Boven and Gilovich, 2003; Van Boven, 2005; Kumar and Gilovich, 2015). Furthermore, experiences are also more likely to be shared with others, whereas possessions may be more prone to use solitarily (Caprariello and Reis, 2013). Experiential purchases activities are typically carried out by groups instead of individuals to relieve the perception of loneliness.

On the other hand, experiential purchases are more often considered as social motivation for talking and also more conducive to conversation value than material purchases (Bastos and Brucks, 2017). Material purchases are more often considered to be driven by external motivation, such as social status and reputation, etc., while experiential purchases are driven mainly by internal motivation and the inner heart (Van Boven et al., 2010). As a result, the conversation of material purchases is more often considered to be showing off and leads to negative social comparison, thus primes the negative stereotype of material purchases (Van Boven, 2005; Carter and Gilovich, 2010). Conversely, experience is more related to personal feelings, which is unique and less comparable than material. Individuals have a high propensity for obtaining positive emotion and social connection from social groups when sharing experiences (Van Boven et al., 2010). The promotion of interpersonal relationships generated from the sharing of experiences is greatly indispensable for the relief of loneliness. In addition, Howell and Hill (2009) also documented that experiential purchases of individuals can effectively enhance the intimacy between them so as to satisfy the basic psychological need of social relatedness. Therefore, we discern that the promotion of social relations may also play an important role in the influence of experiential purchases on loneliness. Compared with material purchases, experiential purchases can better contribute to the relatedness needs and further alleviate a sense of loneliness. Formally, we propose the following hypotheses:

H1: Compared with material purchases, experiential purchases can more effectively alleviate loneliness.

H2: Relational enhancement mediates the effect of experiential purchases on loneliness.

## Study 1

Study 1 aimed to provide initial evidence for our proposition regarding the relationship between the different purchase types (experiential purchases vs. material purchases) and loneliness. Loneliness can be discussed as a stable personality trait or as a temporary state tied to a specific event or circumstance (Cacioppo et al., 2009). Primed loneliness was selected as the measurement for the perceived loneliness. Specifically, we examined if participants who had been primed with loneliness (Maner and Gerend, 2007) and have experiential purchases in retrospect would currently feel less lonely, compared with the individuals with material purchases in retrospect. Referring to the previous studies (Van Boven and Gilovich, 2003), the participants were asked to recall their past experiential or material purchases in their daily activities. After that, the current loneliness was measured. Considering the series of research that has documented experiential purchases may elicit higher happiness than material purchases (Van Boven and Gilovich, 2003; Carter and Gilovich, 2012; Bastos and Brucks, 2017), Study 1 also measured happiness.

### Method

Two hundred and thirty-one undergraduates (109 males; average age = 20.99) from a university in southern China participated in the experiment for monetary rewards. We recruited the participants through WeChat platform, which is the social media with the largest number of users in China. Two (loneliness primed: high vs. low) by two (purchase types: experiential recall vs. material recall) between-group designs were implemented in Study 1; the participants were randomly assigned to either of these conditions. In the high loneliness condition, the recall paradigm was used to manipulate individual affect prime (Cacioppo et al., 2006); the participants read the following scenario: please recall a moment or an event that once made you feel very lonely, for example, you feel that you are isolated, do not have friends or nobody cares about you. In the low loneliness condition, please recall a moment or an event that once made you feel very belonging, maybe you are a member of a big family, or maybe you have a good friend who can share everything. Then, the participants were asked to immerse themselves and write about specific feelings. The participants then completed a two-item manipulation check (Zhou et al., 2008), “I am feeling lonely right now,” “At this moment, I feel quite lonely;” 1 = *strongly disagree*, 9 = *strongly agree*.

The participants then also recalled material or experiential purchases they had made in the past, which followed that of Van Boven and Gilovich (2003). The description of the study was mentioned as follows: “Memory is a momentous part of a valuable life. In this study, we hope you can try to recall a good time of experiential purchases (or material purchases) and try to immerse in it.” In the experiential purchases group, the participants were asked to recall the recent experiential purchase involved “spending money with the primary intention of acquiring a life experience—a series of experiences that are personally encountered or lived through.” In the condition of material purchases group, the participants were asked to recall one of their recent material purchases involved “spending money with the primary intention of acquiring a material possession—a tangible object that you obtain and keep in your possession.” To strengthen manipulation in the task, the participants were also asked to write about the amount of money they spent and how long ago the purchase had been made, a procedure adapted from Rucker et al. (2011).

After that, the participants were then asked about their feelings of loneliness: How lonely do you feel now when you look back on the related purchase? The participants were then asked to report the level of perceived loneliness currently related to the recalled purchases with the 10 items (Pieters, 2013) from the RUCLA scale (Russell et al., 1980); some items were also modified to better reflect the current status of the participants. With a nine-point Likert scale (1 = *strongly disagree*; 9 = *strongly agree*) on the statements such as “when you think back to this purchase, did you feel that you lack companionship currently?;” “when you recall this purchase, did you feel there is no one you can turn to currently?” Happiness was also measured with the three items (α = 0.79, Guevarra and Howell, 2015), such as “How much has this purchase contributed to the happiness of your overall life?” Demographic information was also collected.

### Results and Discussion

#### Manipulation Check

Manipulation checks of loneliness types (α = 0.71) were tested first; two-item responses (*r* = 0.68, *p* < 0.001) were combined to form a single index. The result revealed that the high loneliness primed group had a higher level of loneliness (*M* = 5.26, *SD* = 1.76) than that in the low loneliness primed group (*M* = 2.68, *SD* = 1.53), *t*(229) = 11.23, *p* < 0.001, *d* = 1.56. The manipulation of loneliness was supported.

**FIGURE 1:**
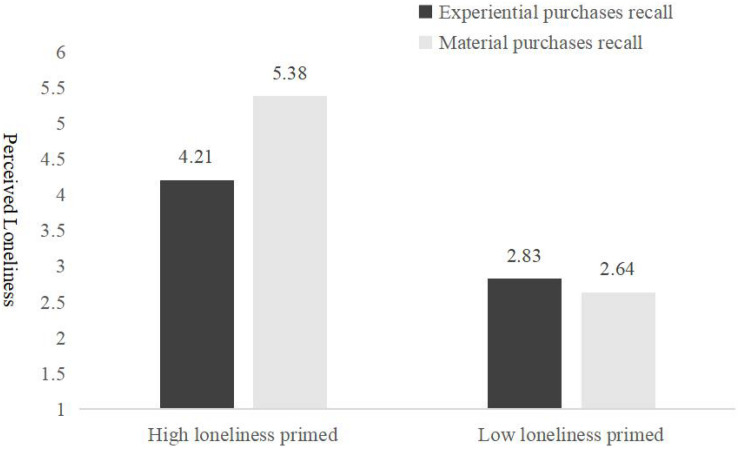
Loneliness perception of experiential and material purchases recall (Experiment 1).

After reversing the scores for the negative items, the total average score of the 10 items (α = 0.87) was used as a variable to measure the loneliness of the participants. The higher the total score, the higher the degree of individual loneliness. 2 (loneliness primed: high vs. low) × 2 (purchase types: experiential purchases recall vs. material purchases recall) covariance analysis of the loneliness of the participants was conducted. The interaction effect between loneliness primed and purchase types has a significant effect on loneliness, *F*(1, 227) = 9.778, *p* = 0.002, η^2^ = 0.04. Furthermore, simple effect analysis revealed that (as shown in Figure 1) in the condition of high loneliness prime, the level of perceived loneliness currently in the experiential purchases group (*M*_*experiential recall*_ = 4.21, *SD* = 0.77) was significantly lower than that in the material purchases group (*M_*material recall*_* = 5.38, *SD* = 1.03), *F*(1, 227) = 13.178, *p* < 0.001, η^2^ = 0.05, while there was no significant difference between the perceived loneliness of experiential purchases (*M_*experiential recall*_* = 2.83, *SD* = 0.77) and material purchases of an individual (*M_*material recall*_* = 2.64, SD = 0.87) when low loneliness was primed, *F*(1, 227) = 0.61, *p* = 0.44. We ran another ANOVA for the alternative mechanisms. The participants who recalled experiential purchases reported a higher level of happiness (*M_*experiential recall*_* = 5.17, *SD* = 0.71) than the participants who recalled material purchases (*M_*material recall*_* = 3.95, *SD* = 1.02), *F*(1, 227) = 10.62, *p* = 0.001, η^2^ = 0.05. The effect of purchase types on loneliness still remained significant while we controlled for the level of happiness (*p* = 0.03).

In Study 1, two different types of purchases were manipulated through the recalling paradigm and preliminarily provide evidence to support H1 that experiential purchases can alleviate loneliness more effectively than material purchases. While Study 1 allows loneliness to be tested on a wide range of material goods/experiences participants have chosen, Study 2 intends to manipulate the same product type into two different types of purchases to validate the results of Study 1 and also aimed to address the notion of relational enhancement.

## Study 2

Study 1 provided a preliminary support for the hypothesis that experiential purchases can more effectively alleviate loneliness than material purchases through the retrospect paradigm. To provide more controlled evidence for our hypothesis, the framing paradigm was conducted and the same product was framed into two different purchase types (experiential vs. material). Framing is an important strategy of experiential marketing involving effective communication, which does not involve the changes of any aspect of the product itself but only how it is portrayed. As a result, experiential framing can be understood as an attempt of marketers to position their products, wherever they may lay on the experiential end of the product continuum—manifested as experiential purchases (Gallo et al., 2019). Therefore, consumers may identify the experiential component of a product and tend to purchase the product for its experiential benefits with the specific product framed as “experiential,” which is referred to as the “experiential purchase.” Relatively, consumers may derive the preservation value from the purchase with the specific product framed as “material,” which is referred to as “the material purchase.” Accordingly, Experiment 2 referred to Mann and Gilovich (2016); the same 3D TV was reframed as either experience or material purchases, respectively. The participants were then immersed in a specific purchase situation and reported their specific feelings. Study 2 was conducted to further test the validity of Study 1 and to shed light on the mechanism underlying our findings—the mediation effect of relational enhancement.

### Method

Two (loneliness prime: high vs. low) by two (framing types: experiential vs. material) between-group design was implemented in Study 2; 284 undergraduates (129 males, average age = 20.4) from a university in southern China participated in the experiment. When the participants completed the experiment task, they were given a small gift for their participation.

First, the retrospect paradigm was used to manipulate affect prime of individuals (Cacioppo et al., 2006); the participants were randomly assigned to the high-loneliness primed or low-loneliness primed group, the same procedure as Study1. And the participants were also asked to write down the specific events and their feelings in detail (Rucker et al., 2011) to strengthen the manipulation. Next, the participants completed a two-item manipulation check on loneliness (Zhou et al., 2008), “I am feeling lonely right now,” “At this moment, I feel quite lonely;” 1 = *strongly disagree*, 9 = *strongly agree*.

Next, a specific type of purchase was manipulated into two different types of purchases. The same 3D TV was reframed into two different types of purchase in terms of experience or material. To encourage the enrollment of 3D TV owners, the description mentioned that, “After we just finished shopping, we always like to imagine and think about the item we just bought.” The participants were asked to imagine that they had just bought a new 3DTV, and it would take a few minutes to think about this. The following description was given to encourage the participants to imagine the purchase types that were framed to them.

The participants in the material framing condition read: “TV is something that people will keep for a period of time. Of course, when you buy it, your goal is to hold it as long as possible, and you like it. Please recall some details of the object, such as product quality, appearance, and performance, just to ensure that you pay attention to all aspects of the object. Please try to consider the specific characteristics of the object and what it feels like to have it.”

The participants in the experiential framing condition read: “TV is something that people use for a period of time. Of course, when you buy it, your goal is to use it better. You like the experience when you use it. Please recall some details of that experience, the experience of watching and specific feelings. Make sure you focus on all aspects of the experience. Please try to consider the specific characteristics of the experience and what it looks like.”

After imagining the purchase scenarios, the participants were then asked about their preference for the 3D TV with a nine-point Likert scale (1 = *very dislike*; 9 = *very like*), and rated how experiential or material they felt about the TV (1 = *purely material*, 9 = *purely experiential*) for manipulation check. Subsequently, loneliness and relational enhancement were also measured. The participants were then asked to report the level of loneliness related to the purchases as in Study 1 (Pieters, 2013). Some items were also adjusted to the experimental situation, with a nine-point Likert scale (1 = *strongly disagree*; 9 = *strongly agree*) on the statements such as “When you think about this 3D TV that you bought, did you feel that you lacked companionship?” and “When you think about this 3D TV that you bought, did you feel there was no one you could turn to?” The relational enhancement was measured through four items (Razavi et al., 2019), with a nine-point Likert scale (1 = *strongly disagree*; 9 = *strongly agree*) on the statements such as “This purchase made me feel more connected to people;” “This purchase helped me make new friends or strengthen existing friendships.” As materialism and loneliness are engaged in bidirectional relationships over time, materialism will also contribute to loneliness (Pieters, 2013). We also measured materialism. The measurement of the materialism was adapted from the short version of materialism scale (six items, Richins, 2004), such as “The things I own say a lot about how well I’m doing in life.” and “I’d be happier if I could afford to buy more things.” Lastly, the participants were also asked to provide the basic demographic information.

### Results and Discussion

#### Manipulation Check

We first tested the manipulation of loneliness primed (α = 0.85); two-item responses (*r* = 0.68, *p* < 0.001) were combined to form a single index. Independent samples *t*-test showed that the high loneliness primed group had a significantly higher level of loneliness (*M* = 5.09, *SD* = 1.76) than that in the low loneliness primed group (*M* = 3.01, *SD* = 1.53), *t*(282) = 8.12, *p* < 0.001, *d* = 1.26. In addition, the participants rated the experiential framing (*M* = 4.92, *SD* = 1.41) TV as more experiential than the material framing TV [*M* = 3.78, *SD* = 1.24; *t*(282) = 6.84, *p* < 0.001, *d* = 0.86]. The TV did not differ in rated favorability, *t*(282) = 0.44, *p* = 0.93. The results reveal that both loneliness prime and purchases types were successfully manipulated.

**FIGURE 2:**
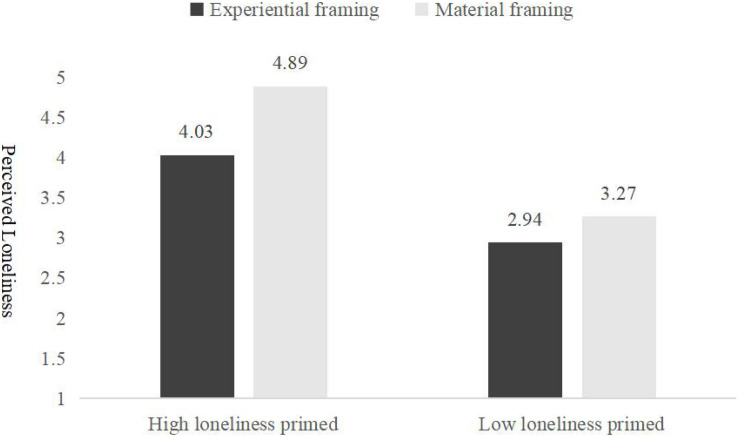
Loneliness perception of experiential and material purchases framing (Experiment 2).

After reversing scores for the negative items, we averaged the responses of the participants on the 10 items for measuring loneliness to create a total score (α = 0.77). With the perceived loneliness (α = 0.69) as the dependent variable, materialism as the control variable [(α = 0.69), a 2 (loneliness primed: high vs. low] × 2 (framing types: experiential purchases vs. material purchases) ANOVA revealed a significant interaction effect, *F*(1,279) = 9.85, *p* = 0.002, η^2^ = 0.03), which indicates that different purchase types have significant differences on loneliness under different levels. The further simple effect analysis show that the rated loneliness level of the participants that were assigned to the experiential framing TV (*M_*experiential framing*_* = 4.03, *SD* = 1.16) was significantly lower than that of the participants that were assigned to the material framing TV (*M_*material framing*_* = 4.89, *SD* = 1.42), *F*(1,279) = 10.55, *p* = 0.001, η^2^ = 0.05, which further supported H1, as Figure 2 shows. For the participants that were manipulated in the low loneliness primed condition, there was no significant difference between the experiential framing group and the material framing group, *F*(1,279) = 1.434, *p* = 0.23, *M_*experiential framing*_* = 2.94, *M_*material framing*_* = 3.27, η^2^ = 0.01.

**FIGURE 3:**
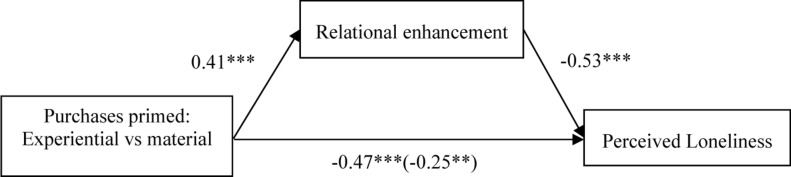
Mediation analysis (in high loneliness primed condition): relational enhancement as a mediator (Experiment 2). *Note* *p-values < 0.05; **p-values < 0.01; ***p-values < 0.001; NS, non-significant.

**FIGURE 4:**
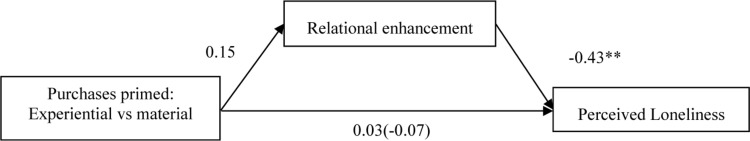
Mediation analysis (in low loneliness primed condition): relational enhancement as a mediator (Experiment 2). *Note* *p-values < 0.05; **p-values < 0.01; ***p-values < 0.001; NS, non-significant.

The Bootstrap method was used to test the mediating effect of relational enhancement (Hayes, 2013; Model 8). More precisely, the bootstrap analysis included framing types (experiential purchases vs. material purchases) as the independent variables, relational enhancement as the mediator variable (α = 0.76), and loneliness as the dependent variable (α = 0.84), loneliness primed as moderator variables. The sample size is 5,000, with a 95% confidence interval (CI). The results showed that a 95% CI for the indirect effect was significant, β = −0.12 (*LLCI* = 0.1421, *ULUI* = 0.5512, excluded 0), indicating that the mediation effect of relational enhancement between types of purchased prime and perceived loneliness was significant. To be specific, relational enhancement mediates the effect of different framing purchases (framing types: experiential purchases vs. material purchases) on loneliness in the high loneliness prime condition (*LLCI* = −0.4132, *ULUI* = −0.1261, excluded 0), as Figure 3 shows. While the mediation effect of relational enhancement did not reveal in the low loneliness prime condition (*LLCI* = −0.0474, *ULUI* = 0.2078, included 0), as Figure 4 shows. The result reveals that the relational enhancement partially mediated the relationship between purchase types and loneliness; H2 was supported.

## Social Nature as Boundary Condition

The above studies provide preliminary evidence for experiential purchases to alleviate individual loneliness more effectively due to the promotion of relational enhancement. Experiential purchases are more likely to share with others, while material purchases are more likely to be used alone (Caprariello and Reis, 2013). When we make and recall the experiential purchases, we feel more frequent social interaction and connection (Van Boven and Gilovich, 2003; Van Boven, 2005; Bastos and Brucks, 2017). Inherent with the social relationship attributes, experiential purchases contribute more conducive to the satisfaction of individual social needs and ease loneliness. Since the relational enhancement embedded in social contact is an essential attribute for the alleviation of loneliness, what if the experiential purchase is deprived of social attributes?

We proposed that social experiential purchases should be more valuable to the solitary experiential purchases; deprivation of sociability of experiential purchases becomes less valuable for the alleviation of loneliness. On the one hand, the need for belonging embedded in social relations is an indispensable element for the alleviation of loneliness. The need for belonging is a basic need of human beings (Baumeister and Leary, 1995) and is also a cornerstone of happiness for individual survival. People satisfy this need mainly by establishing social connections and maintaining close relationships with others (Yamaguchi et al., 2016). Social contact effectively helps to weaken the sense of loneliness, such as, socially, excluded individuals may seek social contact to reduce loneliness and regain the need for belonging. Some evidence also revealed that the social impact of experiential purchases may be more valuable and have more essence than the purchase of experience itself (Carter and Gilovich, 2012; Razavi et al., 2019). Howell et al. (2012) also claimed that experiential purchases facilitate psychological need satisfaction, which contributes to the promotion of subjective well-being. On the other hand, social sharing also serves as an essential feature for experiential purchases, while solitary experiential purchases restrain social interaction and may be less valuable than experiences involving others. In the meantime, the feeling of connection tends to be amplified and bring more valuable experience during the interactive context. By extension, experiential purchases not involving others are likely to be seen as less valuable and, perhaps, less advantageous than material possessions (Caprariello and Reis, 2013). Thus, we predict that the social nature of the experience may serve as an impartible boundary for the effect of experiential purchases on alleviating loneliness. For the solitary experience, the effect of experiential purchases on alleviating individual loneliness will be weaker. Formally, we propose the following hypothesis:

H3: Social nature moderated the effect of experiential purchases on loneliness. For social-experiential purchases, the alleviating effect of experiential purchases on loneliness is stronger, whereas, for solitary-experiential purchases, the effect is weaker.

## Study 3

Study 3 aims to provide support for H3 that social nature moderates the effect of experiential purchases on loneliness. Referring to Caprariello and Reis (2013), the manipulation on experiential purchases with social nature were divided into three groups by emphasizing the social attributes: the social experiential purchases group (by emphasizing the purchase with other people), the solitary experiential purchases group (by emphasizing the purchase alone), and the control group (without any emphasis). Thus, this study took on a 2 (loneliness prime: high vs. low) × 3 (experiential purchases: social vs. solitary vs. control) between-subject design: six comparison sets were conducted to explore the moderating effect of social nature. As a manifestation of adaptation, individuals may have different preferences for solitude (Burger, 1995; Cramer and Cramer, 1998). Individuals with a high preference for solitude may consider solitude as a means of self-regulation, emotional calm, and problem-solving (Caprariello and Reis, 2013). Accordingly, as preference for solitude is relatively independent of social needs, we also measured that as the control variable.

### Method

We recruited 263 participants (118 males) through Qualtrics Panel. The sample came from undergraduate and MBA students at a university in southern China. The average age was 29.24 years (*SD* = 3.24). The participants were randomly assigned to one of the six specific conditions. Loneliness is activated in the same way as Studies 1 and 2. In order to immerse the participants in the experiment effectively, the following guidelines were used: The participants were informed that “Discretionary money refers to money spent with the intent of furthering your happiness. We are interested in how you spend your discretionary money that excludes money spent on needs and everyday necessities (e.g., toiletries and utility bills). We would like you to answer the questions that follow for money that you spent on something discretionary.”

The participants were then randomly assigned to one of the three conditions (experiential purchases: social vs. solitary vs. control). In the social experiential group, the participants read as follows: Please recall the last time that you were spending money with the primary intention of acquiring or participating in a life experience with at least one person. The main focus of this spending should be an activity or experience with another person, rather than buying something that could be kept. You may possibly pay for an outdoor trip with some people, a movie with some people, or a massage together with some people. In the solitary experiential group, the participants read as follows: Please recall the last time that you were spending money with the primary intention of participating in a life experience on your own. The main focus of this spending should be on an activity by yourself, rather than buying something that could be kept. You may possibly pay for an outdoor trip on your own, a movie or a massage. While, in the control group, the participants read as follows: Please recall the last time that you spent money with the primary intention to acquire a life experience. The main focus of this spending should be on an activity, rather than buying something that could be kept. You may possibly pay for an outdoor trip, a movie, or a massage. To strengthen the feeling of such an experience, the participants were also asked to imagine specific situations and write down their experiences.

Subsequently, the participants rated their happiness with the purchases (“I am happy with the experience I have purchased”) and their satisfaction with the purchases (“The money spent on the experience was well worth it”); *1 = totally disagree*, *9 = totally agree*. After that, the participants were then asked to rate loneliness: When you think back to this purchase, what is your current level of loneliness? The participants were then asked to report the level of loneliness related to the recalled purchases with the 10 items (Pieters, 2013) as in Study 1 and Study 2. The participants then indicated the amount of money they spent and how long ago the purchase had been made. After that, the participants completed the measurement of preference for solitude. Referring to Caprariello and Reis (2013), two questions were used to assess the agreement of the participants to preference for solitude. One of the items reflected on the time spent alone, and the other reflected on the time spent with others, such as “I try to arrange my day so that I always have some time of my own” with “I try to arrange my day so that I can do something with someone.” The participants then rated their relative identification in the continuous measurement anchored at 1 (The first statement sounds a lot more like me than the second), 4 (Both statements sound equally like me), and 7 (The second statement sounds a lot more like me than the first). Finally, the participants completed measures of demographic variables.

### Results and Discussion

We invited a researcher to read the narratives of the participants. Unreasonable samples were excluded according to the following methods, not completing the description of the experiential purchases, some non-experiential purchases (such as vehicle maintenance and repair). We also excluded some confusion in solitary purchases or social purchases. Overall, 17 samples were excluded; a total of 246 people were included in the final analysis. The exclusion rates did not yield difference across samples, χ^2^ (d*f* = 2, *N* = 17) = 1.02, *p* = 0.59.

The manipulation of loneliness types was examined afterward (α = 0.85). Independent-samples *t*-test showed that the high loneliness primed group had a significantly higher level of loneliness (*M* = 4.82, *SD* = 1.34) than that in the low loneliness primed group (*M* = 3.46, *SD* = 1.51), *t*(244) = 7.30, *p* < 0.001, *d* = 0.93. Three kinds of experiential purchases did not yield difference in the rating on happiness, *F*(2, 243) = 1.16, *p* = 0.32, also in the rating on satisfaction with related purchase, *F*(2, 243) = 0.08, *p* = 0.92.

After reversing scores for the negative items, the responses of the participants on the 10 items for measuring loneliness were averaged to create the total score. With perceived loneliness as the dependent variable (α = 0.71), preference for solitude as the control variable (α = 0.81), a 2 (loneliness prime: high vs. low) × 3 (experiential purchases: social vs. solitary vs. control) ANOVA revealed a significant interaction effect, *F*(2, 239) = 8.29, *p* < 0.001, η^2^ = 0.07, which indicates that different types of purchases have significant differences in perceived loneliness under different levels.

**FIGURE 5:**
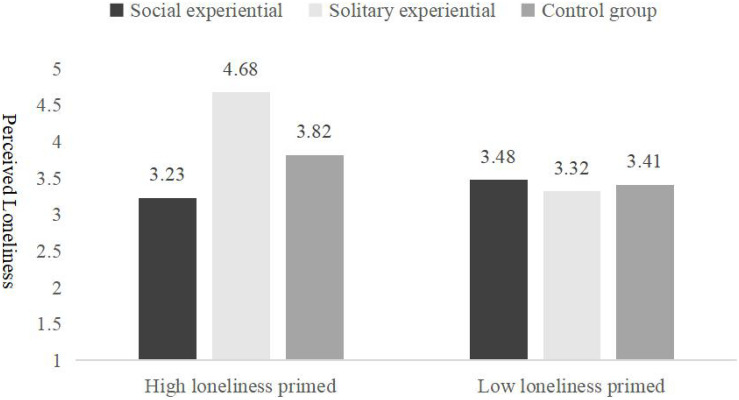
Loneliness perception from experiential and material purchases by social nature (Experiment 3).

Furthermore, simple effect analysis shows that the rated perceived loneliness of the participants who were assigned to the social experiential purchases recall group (*M_*social experiential*_* = 3.23, *SD* = 1.09) … was significantly lower than that of the participants who were assigned to the solitary experiential purchases (*M_*social experiential*_* = 4.68, *SD* = 1.51) and assigned to the control group. (*M_*control group*_* = 3.82, *SD* = 1.60), *F*(1,239) = 14.49, *p* < 0.001, η^2^ = 0.11, as Figure 5 shows. For the participants that were manipulated in the low loneliness prime condition, there was no significant difference between the three groups, *M_*social experiential*_* = 3.48, *M_*solitary experiential*_* = 3.32, *M_*control group*_* = 3.41, *F*(1,191) = 0.15, *p* = 0.86. This result reveals that, for the high loneliness group, social experiential purchases have a higher impact on loneliness alleviation than that in the solitary experiential purchases group. Even the control group has a higher alleviation effort for loneliness than the solitary experiential purchases. We conjecture that, since experiential purchases essentially are more socially present (Caprariello and Reis, 2013; Razavi et al., 2019), solitary experiential purchases highlight inconsistent expectations of individuals and lead to a weaker effect on alleviating feelings of loneliness. For example, as travel occurs more often between groups, individuals may, therefore, feel more isolated when they travel alone. Social nature plays an important role in the effect of experiential purchases to alleviate loneliness, which provides support for H3, and also reinforced H1 and H2.

## General Discussion

In recent decades, the effect of experiential purchase on happiness has attracted large numbers of researchers (Dunn et al., 2011; Guevarra and Howell, 2015; Gilovich and Gallo, 2019), while this study explores the positive effect of experiential purchases on loneliness from another perspective. This study examined the alleviative effect of experiential purchases on loneliness, its internal mechanism, and also the boundary condition of social nature. Specifically, the embedded sociality of experiential purchases contributes to the satisfaction of the social relationship for individuals, thus providing the possibility to alleviate individual loneliness. Social experiential purchases will lead to a higher degree of relief toward loneliness, while the mediation effect of relational enhancement was also supported. Relatively, for the solitary experiential purchases, the alleviating effect of experiential purchases on loneliness is less tight. This effect was robust across people who recall the relative experiential or material purchases (Study 1 and Study 3) or people who imagine that they had just made a relevant purchase (Study 2), as well as controlling for happiness (Study 1) and preference for loneliness (Study 3).

### Theoretical Contributions

Our findings provide insight into an important, real-life consumer-issue loneliness and the ways to reduce it, which has received limited attention from consumer researchers.

On the one hand, the current research reveals a new finding that experiential purchases has a positive effect on relieving negative emotions such as loneliness. Previous studies mainly explored the effect of experiential purchases on positive emotion (such as happiness) and illustrated the effect through parsing, such as providing conversational value (Bastos and Brucks, 2017) and avoiding unfavorable social comparisons (Gilovich and Gallo, 2019). However, the negative emotions receive limited attention. Focusing on the perspective of negative emotions, this study examines the effect of experiential purchases on loneliness, which is an important supplement for previous research. It is worth noting that previous researches have also examined the relationship between experiential purchases and negative emotions. For example, Carter and Gilovich (2010) claimed that material purchases are more comparable and thus more likely to trigger jealousy than experiential purchases. Lin (2018) also documented experiential purchases are more likely to trigger benign envy than material purchases and beneficial to the purchase intention of the envied object. Addressed to the stream of research on experiential purchases associated with negative emotion, our finding provides new strategies on how to cope with the negative impact of loneliness.

On the other hand, this study is a supplement and extension for research on loneliness. Plenty of literature have explicitly or implicitly shown that providing social support and increasing opportunities for social interaction are effective interventions for loneliness (Hawkley and Cacioppo, 2010; Cacioppo et al., 2015; Killgore et al., 2020). Although loneliness can be alleviated or reduced through cognitive and social ways (Cacioppo et al., 2015), there is limited research that has considered the coping strategies from the marketing and consumption behavior perspectives. The inherent purpose of purchase behavior is unique to social interaction, which may have an endogenous impact on individual emotion, just as Killgore et al. (2020) claimed that shared experience has an important effect on alleviating perceived loneliness and restoring happiness. Focusing on the essential attributes of social interaction of experiential purchases and material purchases, the current research explores the effect of experiential purchases on loneliness from several common product types for daily consumption of consumers and enriches the study of loneliness interventions in consumption scenarios.

In addition, the current research proposes and validates an important mediation mechanism with previous research. Experiential purchases first lead to the enhancement of relatedness satisfaction, and then lead to an increase in vitality and ultimately predict subjective well-being (Howell and Hill, 2009). One non-negligible finding of this study is that experiential purchases induce a higher level of relational enhancement, which supports and extends the results of previous research. As a boundary condition for the impact of experiential purchases on loneliness, social nature also further supports the role of relationship enhancement in the alleviation of loneliness through experiential purchases (Caprariello and Reis, 2013; Razavi et al., 2019).

### Implication

With the prevalence of experience economy, the current research is of great significance to the marketing practice of enterprises and also to the intervention of loneliness for psychologists and policymakers.

The current research has some non-negligible implications for the mutual fit between marketing practices and loneliness groups. For example, enterprises can capture negative emotions, such as individual loneliness through various resources in their communities, i.e., social media. Once the lonely consumer is targeted, more experiential purchases practice and promotion are required to better meet the psychological needs of the lonely group. Lonely consumers may have higher emotional preferences for products that emphasize experiences, thus find the products more appealing to them. It is not only beneficial to specific product marketing but also significant to alleviate loneliness. In the meantime, the promotional advertisement can be more appealing if the content emphasizes a great sense of relational enhancement or understates the importance of social nature, thereby enhancing a dominant role of experiential content. As for the promotion strategies and action plans of experiential products, the companies can explicitly highlight emotional and experiential benefits of the products for consumers who suffer from social exclusion and loneliness, such as lovelorn people and job seekers who have been rejected.

Moreover, in addition to the enlightening role of marketing, this study has a wide range of social significance. A study executed by the American Association of Retired Persons found that 35% of adults aged 45 and over felt lonely, compared with 20% 10 years ago (Wilson and Moulton, 2010). Nowadays, loneliness is increasingly becoming a serious social problem. Loneliness affects every individual as well as society. It is a realistic proposition for policymakers and psychologists to pay attention and alleviate individual loneliness (Deckx et al., 2018). Our research shows that experiential purchases are a more obvious way to alleviate loneliness than material purchases, and the social nature serves as a steady mediator. Furthermore, since the social nature of experience is helpful to alleviate loneliness, what if the experience is shared on social media and the loneliness-alleviating reactions from friends? In the future, social policymaking or psychotherapy, it is especially important to focus more on the experience sharing and also the inherently social nature.

### Limitations and Future Research

Consumers derive more happiness from experiential purchases than from material purchases (Van Boven and Gilovich, 2003; Caprariello and Reis, 2013; Bastos and Brucks, 2017), while there is limited research on the relationship between experiential purchases and negative emotions. Through three experiments, the paradigm of recall and scenario simulation was used to verify that individuals feel less lonely after experiential purchases compared with material purchases. However, there are still some limitations and shortcomings in this study, which also need to be further promoted in the future.

First, considering the convenience of sampling and the cost of the experiment, the samples used in this study were mainly college students instead of general populations of consumers. In the meantime, we mainly focused on the topic of alleviation, and neutral loneliness was not adequately set in the experimental design, which may limit the strength of the finding. Future research can expand the scope of the sampling groups to enhance the universality/statistical power of the related research and also consider neutral loneliness during the experimental processing. Second, this paper explored experiential purchases through recall or situational experiments. Big data and other quantitative methods can be used for further research, such as capturing the impact of purchase types on loneliness and individual emotions on social networks, to further consolidate or extend the findings. Third, when examining the boundary effect of social essence, this paper just considers the experiential purchases as a comparison and does not pay more attention to such situations as social material purchases/solitary material purchases. The effect of social essence on material purchases can be further explored in future research. Fourth, this study is only based on the social attributes embedded in the experience of the purchase and interprets the effect on loneliness. Future research can further explore the significance of other attributes, such as self-concept or a sense of meaning, in alleviating loneliness. Fifth, this study interprets the effect on experiential purchases on loneliness based on the embedded social attributes. Future research may further explore the other potential attribute (similar self-concept or a sense of meaning) in alleviating loneliness, which may enrich the stream of research on loneliness.

Last but not least, the current research indicates that experiential purchases are more conducive to alleviate loneliness, while other negative emotions have not been further explored. Loneliness is only one kind of negative emotion, and other similar negative emotions, such as anxiety and depression, can be derived from this study that can be further explored in future research. Furthermore, many researchers have paid attention to existential loneliness in recent years. Existential loneliness is preliminarily defined as a direct consciousness of a sense of isolation from others and the world (Bolmsjö et al., 2019). Having nobody to share life with and the lack of meaning were identified as related to meanings of existential loneliness, which synthesized into a comprehensive understanding of existential loneliness as “being disconnected from life” (Sjöberg et al., 2018). Individuals may encounter existential loneliness even though they have close social ties and do not suffer subjective loneliness. Recognizing the existence of existential loneliness and endeavoring to find intervention strategies to mitigate such negative experiences require more exploration in the future (Sundström et al., 2018; Bolmsjö et al., 2019). This study preliminary explores the relationship between experiential purchases and subjective loneliness, while also shedding light on the research on existential loneliness.

## Data Availability Statement

The raw data supporting the conclusions of this article will be made available by the authors, without undue reservation.

## Ethics Statement

Ethical review and approval was not required for the study on human participants in accordance with the local legislation and institutional requirements. The patients/participants provided their written informed consent to participate in this study.

## Author Contributions

BY: conceptualization, methodology, software, validation, formal analysis, investigation, resources, data curation, and writing – original draft. HY: conceptualization, methodology, resources, data curation, writing – review and editing, supervision, funding acquisition, and project administration. YY: validation, investigation, resources, and data curation. All authors contributed to the article and approved the submitted version.

## Conflict of Interest

The authors declare that the research was conducted in the absence of any commercial or financial relationships that could be construed as a potential conflict of interest.

## Publisher’s Note

All claims expressed in this article are solely those of the authors and do not necessarily represent those of their affiliated organizations, or those of the publisher, the editors and the reviewers. Any product that may be evaluated in this article, or claim that may be made by its manufacturer, is not guaranteed or endorsed by the publisher.
